# A patient with Parkinson’s disease whose sleep status improved after the introduction of continuous subcutaneous foslevodopa/foscarbidopa infusion

**DOI:** 10.1016/j.prdoa.2024.100292

**Published:** 2024-12-02

**Authors:** Hirotaka Sakuramoto, Hiroaki Fujita, Keitaro Ogaki, Keisuke Suzuki

**Affiliations:** Department of Neurology, Dokkyo Medical University, Japan

**Keywords:** Parkinson’s disease, Continuous subcutaneous foslevodopa/foscarbidopa infusion, Sleep disturbances

## Abstract

Continuous subcutaneous infusion of foslevodopa/foscarbidopa (LDP/CDP) may be effective in improving daytime symptoms in patients with Parkinson’s disease (PD). We report on a PD patient for whom LDP/CDP treatment improved motor symptoms, sleep disturbances and sleep architecture as measured by the mobile two-channel electroencephalography/electrooculography recording system.

## Highlights

• A PD patient with motor fluctuations was treated with continuous infusion of foslevodopa/foscarbidopa (LDP/CDP).
• Sleep status was measured by the mobile two-channel electroencephalography/electrooculography recording system.
• After LDP/CDP treatment, motor symptoms improved and off-time reduced.
• Also, LDP/CDP infusion increased total sleep time, Stage N3 duration and sleep efficiency.

## 1 Introduction

Continuous subcutaneous infusion of foslevodopa/foscarbidopa (LDP/CDP) is a novel therapy recently approved for the treatment of Parkinson's disease (PD) patients with motor complications that improves motor fluctuations through continuous dopaminergic stimulation [1], [2]. This treatment may improve nocturnal motor/non-motor symptoms and sleep disturbances via the continuous 24-hour administration. However, no studies have objectively evaluated the effect of LDP/CDP on sleep status. Here, we report a patient with PD treated with continuous subcutaneous infusion of LDP/CDP who experienced improvement of motor symptoms, sleep disturbances and sleep structure as measured by the mobile two-channel electroencephalography/electrooculography recording system.

## 2 Case report

The patient was a 50-year-old male PD patient with a disease duration of 9 years. The patient had several episodes of dream enactment behavior (DEB) during sleep before the development of motor symptoms. Wearing off began at age 45, and the patient was taking levodopa six times a day since age 48. At age 49, dopamine agonists had to be discontinued because the patient developed severe hallucinations and delusions. Afterward, the dose of the dopaminergic drugs could not be increased owing to worsening of the psychiatric symptoms, and the addition of non-dopaminergic drugs was also ineffective. He had difficulty working due to off-time symptoms despite adjustment of anti-PD medication and was therefore admitted to our hospital for the introduction of LDP/CDP. The patient was on levodopa/carbidopa 600 mg per day before admission. During on time, the patient was able to walk independently, but during off time (15 h a day), he had difficulty standing and could not walk due to freezing of gait despite taking levodopa/carbidopa 6 times a day (100 mg at 7:00, 10:00, 12:00, 15:00, 18:00 and 23:00). Dyskinesia was not detected at the hospital or at home. The patient had a Mini-Mental State Examination score of 30 and a Montreal Cognitive Assessment score of 28. The patient had previously experienced episodes of visual and auditory hallucinations and delusions, which improved with the discontinuation of dopamine agonists, catechol-O-methyltransferase inhibitors and monoamine oxidase B inhibitors. Before and 9 days after the introduction of LDP/CDP, motor symptoms were assessed during the off state with the Movement Disorders Society-Unified PD Rating Scale (MDS-UPDRS) part III (motor examination). Daytime sleepiness was evaluated by the Epworth Sleepiness Scale (ESS), and depressive symptoms were assessed by the Beck Depression Inventory (BDI)-II. The PD Sleep Scale (PDSS)-2 was used to evaluate PD-related sleep problems [3]. Also, the following PDSS-2 domain scores were assessed: “disturbed sleep” (sum of items 1–3, 8, and 14), “motor symptoms at night” (sum of items 4–6, 12, and 13), and “PD symptoms at night” (sum of items 7, 9–11, and 15). A portable recording system with a set of electroencephalogram and electrooculogram leads (Sleepgraph®, Proassist Inc., Japan) [4] was used to assess sleep status before and 9 days after the introduction of LDP/CDP. Sleep stages were determined according to the criteria of the American Academy of Sleep Medicine [5] and manually analyzed by a trained, certified sleep technician. Rapid eye movement sleep without atonia (RWA) was assessed via chin electromyography (EMG) and was defined as: 1) an epoch of REM sleep (Stage R) with at least 50 % of the duration of the epoch having greater than the minimum amplitude demonstrated in NREM sleep or/and 2) at least 50 % of the 3-second mini-epochs contain bursts of transient muscle activity lasting 0.1–5 s, with an amplitude 4 times greater than the background EMG activity. Each analysis was carried out blind to the patient information, except for age, sex and diagnosis of PD.

**Fig. 1:**
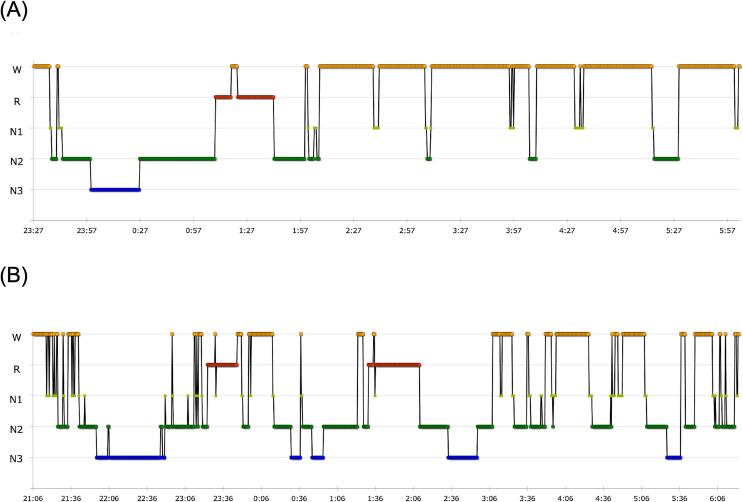
Comparison of hypnograms before and after the introduction of LDP/CDP. Hypnograms obtained from a portable sleep testing system before (A) and after (B) the introduction of LDP/CDP are shown. The horizontal axis indicates time, and the vertical axis indicates sleep stage. W = Stage Wake; R = Stage R (REM sleep); N1 = Stage N1; N2 = Stage N2; N3 = Stage N3.

LDP/CDP was introduced at an infusion rate of 0.17 mL/h (28.9 mg/h) during the day [2] and 0.15 mL/h during the night. Nine days later, the patient’s MDS-UPDRS part III score improved from 63 to 48 points, and the off time decreased from 15 h to 6 h. Also, the PDSS-2 total score, PDSS-2 domain scores, ESS and BDI-II scores improved (Table 1). Analysis via a portable sleep testing system performed 9 days after the introduction of LDP/CDP revealed an increase in total sleep time, a decrease in wake after sleep onset time and an increase in sleep efficiency from baseline (Table 1). RWA was observed for 1.5 min (5.1 % of Stage R) before LDP/CDP treatment and for 6.5 min (10.6 % of Stage R) after LDP/CDP treatment. RWA observed on chin EMG during REM sleep and a history of DEB episodes were compatible with comorbid REM sleep behavior disorder. Moreover, the number of rapid eye movement (REM) sleep episodes and the duration of Stage N3 increased, and the hypnogram showed marked improvement in sleep architecture after LDP/CDP treatment (Fig. 1).

**Table 1:** Comparison of clinical parameters before and after the introduction of LDP/CDP.

	Before LDP/CDP introduction	After LDP/CDP introduction
MDS-UPDRS III	63	48
Off-time (h)	15	6
PDSS-2 total score	41	14
PDSS-2 domain score		
Disturbed sleep	13	9
Motor symptoms at night	16	3
PD symptoms at night	12	2
ESS	13	9
BDI-II	10	3
Sleep parameters		
TIB (min)	471.5	576.5
TST (min)	179.0	412.0
Sleep latency (min)	9.0	10.5
WASO (min)	208.0	133.5
Sleep efficiency (%)	38.0	71.5
Sleep stages, min (% of TST)		
Stage R, min (%)	29.5 (16.5)	61.5 (14.9)
Stage N1, min (%)	18.5 (10.3)	37.0 (9.0)
Stage N2, min (%)	103.5 (57.8)	211.5 (51.3)
Stage N3, min (%)	27.5 (15.4)	102.0 (24.8)
RWA, min (%)	1.5 (0.8)	6.5 (1.6)
RWA / stage R (%)	5.1	10.6

MDS-UPDRS III = Movement Disorders Society-Unified PD Rating Scale part III; PDSS-2 = PD Sleep Scale-2; ESS = Epworth Sleepiness Scale; BDI-II = Beck Depression Inventory-II; TIB = time in bed, TST = total sleep time, WASO = wake after sleep onset, Stage W = wakefulness, Stages N1-N3 = non-REM sleep, Stage R = REM sleep; RWA = REM sleep without atonia. Sleep efficiency (%) = (total sleep time (min)/time in bed (min)) × 100.

## 3 Discussion

In our patient, LDP/CDP treatment increased total sleep time, Stage N3 duration and sleep efficiency and decreased wake after sleep onset time. Notably, improvements in sleep architecture and sleep quality related to the marked increase in the proportion of deep sleep (Stage N3) in total sleep time and decreased wake after sleep onset time may have had favorable effects on daytime motor function and sleepiness. Patient satisfaction with sleep was successfully improved, as shown by changes in the PDSS-2 and ESS scores. In a 12-month, phase III study of 244 PD patients with ≥2.5 h of off time/day, LDP/CDP reduced the off time by 3.5 h and also improved sleep disturbances according to the PDSS-2 total and all domain scores [1]. Similarly, in our patient, LDP/CDP treatment resulted in improvement in the PDSS total and all three domain scores, including PD symptoms at night. In a multicenter, randomized, double-blind crossover study consisting of 46 PD patients with insomnia, subcutaneous night-time apomorphine infusion successfully improved sleep quality, sleep onset and maintenance insomnia according to questionnaires [6]. However, in those studies, sleep duration, sleep efficiency and sleep stages were not assessed with objective measures.

Considering that motor activity is reduced during the night and that the physiological circadian rhythm involves a decrease in dopamine secretion during the night, it has been suggested that the nocturnal infusion rate of LDP/CDP may be reduced to 50–80 % of the daytime infusion rate [2]. In the present patient, sleep fragmentation, possibly due to nocturnal off time-related symptoms, was considered significant before treatment, and a nocturnal infusion rate of 88 % of the daytime infusion rate (minimum set infusion rate of 0.15 mL/h) was used.

In our patient, the two-channel portable biopotential recording system was used to evaluate sleep status. The validity of the sleep parameters obtained with this device has been shown to correlate well with those obtained via polysomnography in healthy individuals [4] and in psychiatric disorders [7]. Additionally, Kataoka et al. [8] compared the results of polysomnography and Sleepgraph in eight patients with PD and reported that Sleepgraph had a slightly lower detection rate for N3 stage than polysomnography but was useful for evaluating overall sleep stages and RWA. Our patient’s Sleepgraph was performed twice in the hospital, but this device is easy to use and may be suitable for home use. As suggested by a previous study [8], it may be a possible alternative to polysomnography that can be performed at home for PD patients who have difficulty being admitted to the hospital.

In conclusion, we report the case of a PD patient in whom LDP/CDP administration improved sleep disturbances and sleep architecture by reducing nocturnal off time-related symptoms. LDP/CDP may be an effective treatment for improving nocturnal and early morning off time-related symptoms as well as sleep disturbances.

## Author contributions

All the authors contributed to the interpretation of the data for this study. HS drafted the manuscript. HS, HF, TS, and KO contributed to the acquisition of the data. HF, KO and KS performed critical reviews of important intellectual content. All the authors read and approved the final version of the manuscript.

## CRediT authorship contribution statement

**Hirotaka Sakuramoto:** Writing – original draft, Methodology, Data curation. **Hiroaki Fujita:** Writing – review & editing, Methodology, Conceptualization. **Keitaro Ogaki:** Writing – review & editing, Methodology, Conceptualization. **Keisuke Suzuki:** Writing – review & editing, Supervision, Methodology, Conceptualization.

## Ethics approval

Written informed consent for participation was obtained from the patient. This case study was performed in accordance with the Declaration of Helsinki.

## Funding

The authors received no financial support for the research, authorship, and/or publication of this article.

## Declaration of competing interest

The authors declare the following financial interests/personal relationships which may be considered as potential competing interests: H. Sakuramoto, H. Fujita and K. Suzuki received lecture fees from AbbVie, Inc., outside the submitted work. K. Ogaki has nothing to disclose.
